# Microtubules are necessary for proper Reticulon localization during mitosis

**DOI:** 10.1371/journal.pone.0226327

**Published:** 2019-12-26

**Authors:** Ulises Diaz, Zane J. Bergman, Brittany M. Johnson, Alia R. Edington, Matthew A. de Cruz, Wallace F. Marshall, Blake Riggs

**Affiliations:** 1 Department of Biology, San Francisco State University, San Francisco, California, United States of America; 2 Department of Biochemistry & Biophysics, UCSF Mission Bay, San Francisco, California, United States of America; Institut de Genetique et Developpement de Rennes, FRANCE

## Abstract

During mitosis, the structure of the Endoplasmic Reticulum (ER) displays a dramatic reorganization and remodeling, however, the mechanism driving these changes is poorly understood. Hairpin-containing ER transmembrane proteins that stabilize ER tubules have been identified as possible factors to promote these drastic changes in ER morphology. Recently, the Reticulon and REEP family of ER shaping proteins have been shown to heavily influence ER morphology by driving the formation of ER tubules, which are known for their close proximity with microtubules. Here, we examine the role of microtubules and other cytoskeletal factors in the dynamics of a Drosophila Reticulon, Reticulon-like 1 (Rtnl1), localization to spindle poles during mitosis in the early embryo. At prometaphase, Rtnl1 is enriched to spindle poles just prior to the ER retention motif KDEL, suggesting a possible recruitment role for Rtnl1 in the bulk localization of ER to spindle poles. Using image analysis-based methods and precise temporal injections of cytoskeletal inhibitors in the early syncytial Drosophila embryo, we show that microtubules are necessary for proper Rtnl1 localization to spindles during mitosis. Lastly, we show that astral microtubules, not microfilaments, are necessary for proper Rtnl1 localization to spindle poles, and is largely independent of the minus-end directed motor protein dynein. This work highlights the role of the microtubule cytoskeleton in Rtnl1 localization to spindles during mitosis and sheds light on a pathway towards inheritance of this major organelle.

## Introduction

The organization and dynamics of eukaryotic cells during cell division have been extensively studied over the past several decades with key discoveries made with respect to chromosomal partitioning, and the changes of the cytoskeleton network [1–3]. Lesser understood is the dynamics of the cytoplasmic organelles during cell division. Studies have investigated organelle dynamics during mitosis, including the disassembly and reassembly of the Golgi apparatus [4,5] and the structural changes and localization of the Endoplasmic Reticulum (ER) [6,7]. In addition, there have been several studies in mammalian cells that have focused on the remodeling of ER structure exhibiting a more tubular morphology during mitosis [8,9]. Recent studies have begun to shed light on the regulatory mechanism involved in these dramatic changes of the Golgi [10] and the ER [11]. While there have been several advances of our understanding of organelle dynamics during mitosis (for a detailed review see [12]), several unanswered questions still remain, including the role of the cytoskeletal network during mitosis and the mitotic kinases and motor proteins involved in these dramatic reorganization events.

The microtubule network undergoes a dramatic reorganization event upon entry into mitosis, forming a bipolar spindle responsible for attachment and partitioning of genetic material. This relies on the coordination of several regulatory proteins and pathways involving mitotic kinases, several microtubule associate proteins and both plus-end and minus-end directed motors [13,14]. Earlier studies have shown that the ER shares a close interdependence with microtubules [15,16] but it is unclear whether the mitotic reorganization of the microtubule network contributes a role in these mitotic changes in ER changes morphology or partitioning. Imaging and functional studies show that the ER shares a close association with mitotic spindle poles, and that microtubules and ER are important for nuclear envelope reformation at mitotic exit [7,11,17,18]. However, the mitotic factors that associate and drive ER reorganization and partitioning are poorly understood.

The Reticulons, a family of ER shaping proteins, were initially identified as factors involved in the generation of curvature of the tubular ER network, however several other ER shaping proteins have been recently been characterized [19] including REEPs, Spastin, Atlastin, and DP1 [20,21]. Furthermore, defects in the function of these proteins have been linked to several neurodegenerative diseases including hereditary spastic paraplegias (HSP) and Alzheimer’s disease [22,23].

A recent study has characterized the Drosophila REEPs (Reep A and B) and their role in formation of tubular ER morphology in axons [24]. Additionally, in mammalian cells REEP 3/4 are important for aligning the ER along the periphery of the mitotic spindle and maintaining ER away from chromatin during metaphase [25]. Although the role of Reticulon and REEP proteins in forming ER tubules is well documented [23], there are no quantitative studies that characterize a possible role of microtubules in Reticulon and REEP spindle pole enrichment. In addition, there have been limited studies involving the role of Reticulon family members during mitosis and it is unclear if microtubules are important for their localization [26].

The syncytial divisions of the early Drosophila embryo provide an excellent model to investigate the role of mitotic factors involved in the reorganization and partitioning of the ER during mitosis. Once fertilized, the Drosophila embryo experiences a series of 13 rapid synchronous nuclear divisions before each of the 5,000 nuclei are encapsulated by membrane furrows to form a multicellular embryo [27]. During these syncytial divisions, the first 9 divisions occur in the interior of the embryo, after the 9^th^ division, the nuclei migrate to the cortex of the embryo and go through 3 more rounds of division. These syncytial divisions are rapid and do not have gap phases in their cell cycle, rather only experiencing a S-phase (DNA replication) and mitosis. These cortical nuclear divisions (cycle 10–13) provide an excellent platform to examine mitotic events. Additionally, several studies have demonstrated that the mechanism of syncytial mitosis in the early embryo is comparable to mechanism of mitotic events found in other systems [28–31]. Furthermore, in the syncytial embryo the ER demonstrates a dramatic rearrangement in both structure and localization during mitosis, in line with the mitotic ER changes seen in other systems [11,17].

Here we sought to examine the necessity of key mitotic components in the reorganization of the ER during mitosis. We performed microinjections of small molecule inhibitors targeting the cytoskeleton, major mitotic kinases, and motor proteins known to play a key role in mitotic progression. Using a Drosophila Reticulon, Reticulon-like 1 (Rtnl1), we show that microtubules, not microfilaments, are necessary for reorganization of the ER during mitosis. We also demonstrate that localization of Rtnl1 at the poles relies on a dynamic astral microtubule population and is independent of the minus-end directed motor dynein. In the course of this work, we have developed a quantitative approach to examine ER localization and movement to the spindle poles during mitosis, allowing us to measure changes in the timing and extent of reorganization simultaneously under different perturbations. The results presented here provide an initial framework of the mechanism involved in mitotic ER reorganization and insights into a pathway towards ER inheritance during cell division.

## Materials and methods

### Fly stocks and genetics

His2Av-RFP flies were a gift from Patrick O’Farrell (UCSF, San Francisco, CA).

Rtnl1-GFP[G00199] stock was obtained from the Flytrap database [26]. Lines were crossed with His2Av-RFP, generating genotypes w;His2Av-RFP; Rtnl1-GFP[G00199]. Stocks Y[1] v[1]; P{y[+t7.7] v[+t1.8] = TRIP.JF03177}attP2 (DHC 64 RNAi), y[1] w*; P{w[+mC] = UAS-Lam.GFP}3–3, w[*]; P{w[+mC] = UASpRFP.KDEL}8, P{w[+mC] = out-GAL4::VP16.R}1, w[*]; P{w[+mC] = GAL4-nos.NGT}40; P{w[+mC] = GAL4::VP16-nos.UTR}CG6325[MVD1] (triple maternal driver), w*; M{w[+*] = ReepB-GFP}ZH-86Fb, were obtained from the Bloomington Stock Center (NIH P40OD018537). W; UAS-mCherry tub, was obtained from the laboratory of Patrick O’Farrell. Homozygotes of Rtnl1[G00199]; ReepB-GFP; UAS-mCherry tub; His2Av-RFP or UAS-GFP-Lamin; UAS-RFP-KDEL were created by crossing individual fluorescent lines to balancer stocks and then mating the F1 progeny and driven using the triple maternal driver line for expression in the early embryo.

### Embryo collection and microinjection

Embryos were staged according to criteria established by Campos-Ortega and Hartenstein [32]. Embryos were collected on grape-juice agar plates, aged on collection plates and dechorionated by hand. Dechorionated embryos were briefly desiccated and microinjected as previously described [33]. Needle concentrations for injected solutions were as follows: Colchicine and paclitaxel (Sigma-Aldrich) [5μM], Cytochalasin D (Sigma-Aldrich), Binucleine 2 (Sigma-Aldrich), and BI 2536 (Thermo Fisher) [10μM], Ciliobrevin D (Calbiochem) [100μM]. All drugs were dissolved in DMSO which was limited to a final concentration of 10%. Tubulin HiLyte 488 and 647 (Cytoskeleton Inc.) was injected at 1mg/ml in nuclease free water (New England Biolabs). Microinjection of embryos was performed with ~0.1 μl of RTNL1 dsRNA (1.5μg/μl) injected into the dorsoanterior region of the embryo during the syncytial blastoderm stage.

### Live imaging of Drosophila embryos

For our qualitative approach, embryos were imaged on a Zeiss SD Observer inverted microscope (Zeiss Cell Observer, Carl Zeiss Microimaging, Inc.) with with a C-Apochromat 1.2 NA 100x objective (Carl Zeiss MicroImaging, Inc.), using 488 nm and 543 nm wavelengths from an argon laser. For our quantitative approach, embryos were imaged on an IN Cell Analyzer 6000 high content analysis (HCA) imaging system (GE Healthcare) with a Plan Apochromat Lambda 60X 0.95 NA dry objective lens (Nikon Instruments, Inc.), using a 488 nm diode laser, a 561nm DPSS laser, and a 641 nm diode laser.

### Imaging quantitation

**Fig 1:**
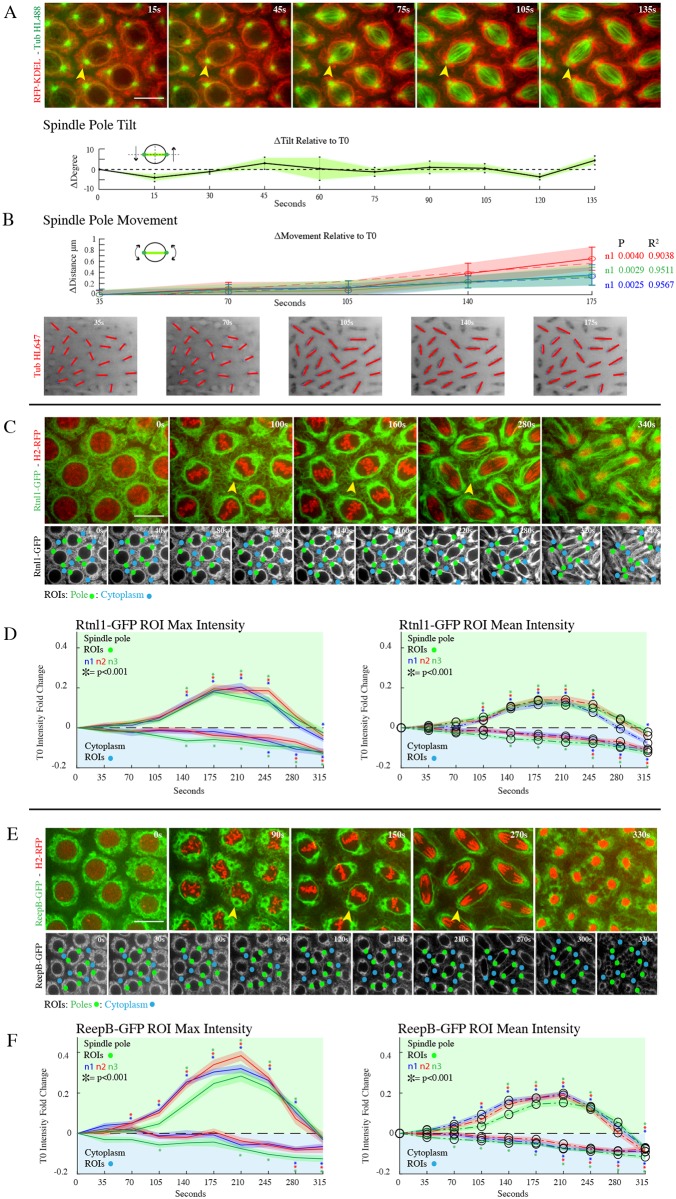
Rtnl1-GFP and ReepB-GFP displays a dramatic reorganization to the spindle poles during mitosis. **(A)** Cycle 11 Drosophila embryo expressing RFP-KDEL (red), microinjected at stage 10 with tubulin HiLyte488 (green). KDEL shows an accumulation to the spindle poles and along the perispindle region during mitotic spindle formation at prometaphase (arrowheads). Spindles show a rocking motion under ±5 degrees around a fixed axis prior to mitosis through metaphase. **(B)** Plot for spindle displacement along nucleus prior to and during mitosis. Tubulin HiLyte 647 was injected into Rtnl1-GFP embryos to measure changes in spindle pole positioning in the [x,y] direction of 0.3 μm relative to centriole position at T0 (n = 3 p = 0.0040, 0.0029, 0.0025 R^2^ = 0.9038, 0.9511, 0.9567) allowing for an accurate estimation for the placement of spindle ROIs during the early stages prior to mitosis where spindles are not yet morphologically visible. **(C)** Cycle 11 Drosophila embryo expressing Rtnl1-GFP (green), and H2Av-RFP (red). Condensation and alignment of chromosomes (H2-RFP) shows the progression of mitosis. At onset of mitosis, an increase in Rtnl1-GFP signal occurs at the nuclear periphery, most notably at the poles (arrowheads). Rtnl1-GFP follows the spindle periphery throughout metaphase and anaphase. It is then divided into the two daughter nuclei with a series of strings between them and bright staining at the midbody. **(D)** Rtnl1-GFP intensity at spindles and cytoplasm are plotted in green and blue lines, respectively Rtnl1-GFP is enriched at spindles and depleted from the cytoplasm, with bulk movement occurring between frames 3–8 (prophase-anaphase). Max and mean spindle intensities peak at a 0.2-fold enrichment compared to T0 (p<0.001 for all samples at T210). **(E)** Cycle 11 Drosophila embryo expressing ReepB-GFP (green), and H2-RFP (red) was imaged throughout mitosis. ReepB-GFP, similar to Rtnl1-GFP in C showed an increase in GFP signal occurs at the nuclear periphery and at the poles (arrowheads). ReepB-GFP follows the spindle periphery throughout metaphase and anaphase and displays bright staining at the midbody.**(F)** ReepB-GFP intensity at the spindle poles is measured using green ROIs, while ReepB-GFP intensity in the cytoplasm is measured using blue ROIs, then plotted with the same green and blue lines as in graph D. Measurements between frames 3–8. Max intensities spindle ROIs peak at a 0.4-fold enrichment compared to T0, and mean interties for those ROIs peak at 0.2-fold enrichment (p<0.001 for all samples at T210).

Rtnl1-GFP and ReepB-GFP enrichment to spindles and depletion from the cytoplasm was quantified using a custom MATLAB script to manually segment ROIs, bulk extract measurements, then track and assemble data for individual ROIs across a series of images. In total, we assembled mean and max intensity data for 7200 ROIs and plot data for 14,400 normalized measurements (Figs 1 & 6). Early ROI spindle positions were determined without a spindle marker by referencing the future locations for spindles from time points when they become morphologically visible. Spindle ROIs for time points where spindles are not morphologically visible include time points 0, 35, and 70 seconds. To validate our method of ROI selection for non-morphologically visible spindles at 0, 35, and 70 seconds, we injected tubulin HiLyte 488 into RFP-KDEL embryos, and tubulin HiLyte 647 into Rtnl1-GFP, to reveal that spindles have very little, insignificant [x,y] movement around the nucleus during these initial time points (Fig 1A & 1B). Furthermore, 20 spindle ROIs and 20 Cytoplasm ROIs are tracked across a series of 10 frames for a minimum of 3 embryos per treatment. In addition, data for all ROIs is normalized against individual ROI “time 0” to produce intensity fold change. Lastly, binary images containing all manual ROI segmentations are saved and can be used to extract and assemble additional measurements. All p-values were determined using an unequal variances t-test for each line and fold change comparison. For a complete description of this program and the functions it uses please refer to the supplemental material (S8 Fig).

## Results

### Rtnl1 and ReepB localize to the perispindle region and spindle poles during mitosis in the early Drosophila embryo

In order to investigate mitotic ER dynamics, we analyzed the movement and reorganization of three ER markers: RFP-KDEL, Rtnl1-GFP, and ReepB-GFP during mitosis of the syncytial blastoderm (cycles 10–13) in the early Drosophila embryo (Fig 1). Embryos expressing an ER retention sequence, KDEL, fused with the red fluorescent-tag RFP (red) were injected with tubulin labeled with HiLyte 488 and imaged during cycle 11 (Fig 1A). We observed an accumulation of ER membrane at the nuclear envelope at prophase. Upon nuclear envelope breakdown (NEB) at prometaphase, RFP-KDEL moved to the perispindle and spindle poles regions. As the mitotic spindle formed, the ER became more focused around spindles during metaphase (Fig 1A, arrowheads).

To employ a quantitative approach in measuring ER membrane movement, we defined regions of interests (ROIs) at spindle poles when they became morphologically visible, then referenced those locations to place ROIs on spindles at timepoints just prior to mitosis when they are not morphologically visible. Past observations, as well as prior studies [34] show a movement of the spindle poles during NEB and spindle formation, however, this movement did not compromise our method for ROI selection since it occurs when spindles are already morphologically visible (Fig 1A and 1B). We examined the movement of spindle poles just prior to the start of mitosis through metaphase in RFP-KDEL and Rtnl1-GFP embryos injected with fluorescently labeled tubulin. Measurements of spindle pole [x,y] orientation in RFP-KDEL embryos injected with Tubulin HiLyte 488 showed a small, rocking tilt of 5 degrees around a central axis, suggesting that spindle position oscillates around a fixed point just prior to mitosis through metaphase (Fig 1A). To test a linear model, we microinjected Tubulin HiLyte 647 into 3 Rtnl1-GFP embryos and quantified spindle movement around the nucleus in reference to T0 (Time point 0) for 20 spindles per embryo (Fig 1B n = 3 p = 0.0040, 0.0029, 0.0025 R^2^ = 0.9038, 0.9511, 0.9567). Prior to NEB, the standard error for the mean spindle movement between frames was under 0.3μm, which is negligible for ROI selection considering we used circular ROIs of 2μm. After NEB through metaphase, there was an increase in spindle movement with a maximum displacement of 0.9 μm, however this is also negligible given that spindles are morphologically visible at this time. Furthermore, the lack of spindle pole movement in the [x,y] direction just prior to mitosis enabled the reliable selection of ROIs up to 70 seconds before they became morphologically visible.

Next, we examined Rtnl1-GFP enrichment to spindle poles and depletion from the cytoplasm. Rtnl1 has been suggested to drive ER membrane changes and promote curvature and tubule formation [19]. The Rtnl1-GFP tagged line was identified in a protein trap screen [35] with the GFP tag being in-frame with the endogenous locus of the reticulon gene. Here, we used Rtn1 as a marker for tubular ER and created a transgenic line expressing both Rtnl1-GFP and the DNA marker, His2Av-RFP (H2-RFP) to monitor the different stages of mitosis. During interphase, Rtnl1-GFP localization was spread throughout the cytoplasm, however as the embryo enters mitosis, Rtnl1-GFP displayed a strong accumulation at the nuclear envelope (Fig 1C, S1 Movie). At prometaphase, as the nuclear envelope destabilized, there was a rapid and focused localization of Rtnl1-GFP at the poles (Fig 1C, arrowheads). Rtnl1-GFP accumulated at the poles during metaphase and was found along the perispindle region similar to what was observed with KDEL. To quantitate these changes, we measured spindle pole enrichment (green dots) and cytoplasmic depletion (blue dots) over successive time frames corresponding to the early stages of mitosis (Fig 1D). Measurements of Rtnl1-GFP at prophase showed a steady increase (T140:T245) in intensity at the spindle pole ROIs (green dots), peaking around 0.2-fold increase for both max and mean intensity measurements. For max intensity measurements, we saw a significant increase (p<0.001) at T140 for all 3 samples. For mean intensity measurements we saw a significant increase (p<0.001) at T105 for all 3 samples. Correspondingly, there is a gradual drop in Rtnl1-GFP intensity at the cytoplasmic ROIs (blue dots) over the same time frame (T140: T315) for max and mean intensity measurements. These results indicated a shift in Rtnl1 localization from the cytoplasm towards the spindle poles at metaphase.

In order to corroborate this result of Rtnl1 movement during the early stages of mitosis, we examined reorganization of the ER shaping protein, ReepB (Fig 1E). Previous studies have described the role of REEPs in organization of the ER during mitosis and their ability to associate with microtubules. Similar to what was observed with Rtnl1, ReepB showed an accumulation at the nuclear envelope during prophase and reorganized to the spindle poles and perispindle region at metaphase. Measurements of ReepB-GFP at prophase showed a steady increase (T140:T245) in intensity at the spindle pole ROIs (green dots), peaking around a 0.4-fold max intensity increase and 0.2-fold mean intensity increase. For max intensity measurements, we saw a significant increase (p<0.001) at T140 for all three samples starting at T70. For mean intensity measurements we saw a significant increase (p<0.001) at T140 for all 3 samples, with two of the samples showing an increase starting at T105, and the third sample showing an increase starting at T70. Again, we measured a gradual drop in ReepB-GFP intensity at cytoplasmic ROIs (blue dots); however, we observed a slightly different time frame. Here we saw max intensity cytoplasmic measurements gradually deplete starting at T210, with mean intensity cytoplasmic measurements depleting starting at T70. These results also indicated a shift in ReepB localization from the cytoplasm towards the spindle poles at metaphase.

### Rtnl1 displays a steady enrichment prior to major reorganization of the ER early in mitosis

**Fig 2:**
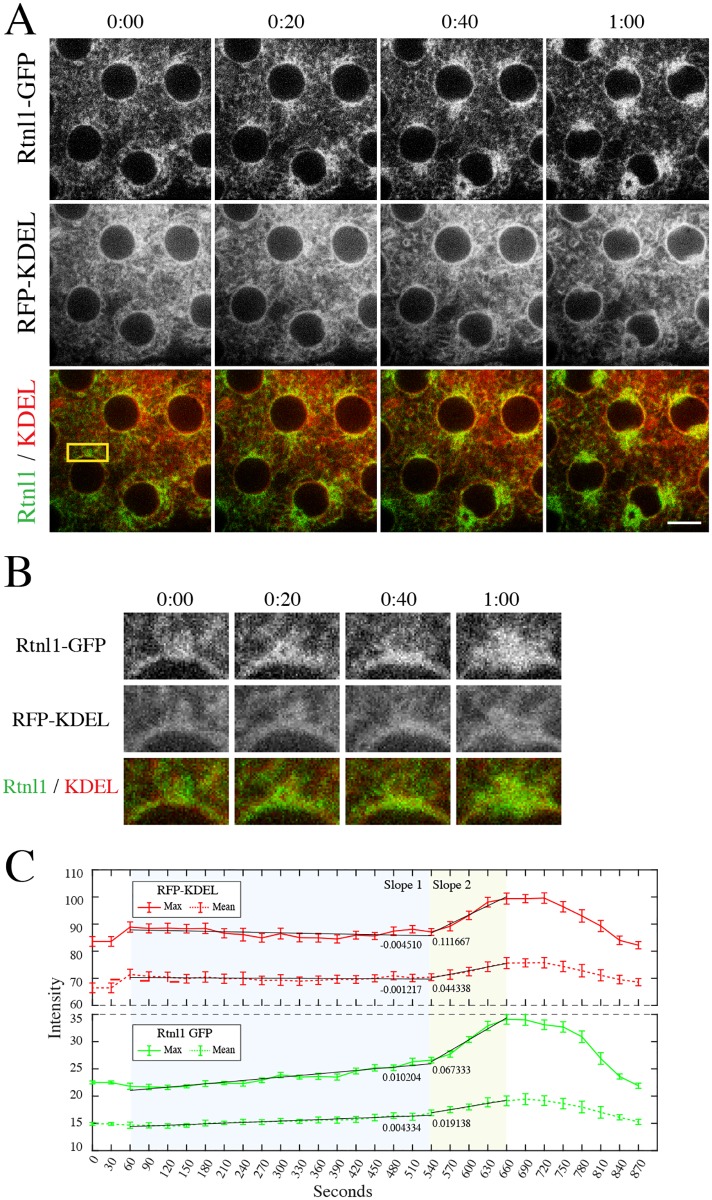
Enrichment of Rtnl1-GFP precedes major reorganization of ER to the poles early in mitosis. **(A)** Stage 10 Drosophila embryo expressing Rtnl1-GFP (green) and RFP-KDEL (red) as it proceeds through one minute during the early stages of mitosis. GFP signal accumulation at the poles arrives and intensifies before the RFP signal, indicating an enrichment of Rtnl1-GFP before an equal amount of ER. **(B)** High-magnification (2x) inset of one of the poles indicated by a box in A. **(C)** Graphs of max and mean fluorescence intensities of RFP-KDEL (red line) and Rtnl1-GFP (green line) of ROI of box from A, throughout mitosis. There is a steady enrichment of Rtnl1-GFP intensity at the ROI from the start of mitosis to NEB at the end of prophase from 0–540 seconds. Bottom graph, slope 1: 0.01024. In contrast, measurement of max and mean intensities of RFP-KDEL showed no increase over the same time points from 0–540 seconds. Top graph, slope 1: -0.004510. N = 3 embryos imaged. Time is in min:sec. Error bars represent standard error. Scale bar is 10μm.

Prior studies have shown that there is a strong accumulation of ER membrane at the poles beginning at prometaphase, after NEB, through metaphase [11,17]. Based on our localization studies (Fig 1), we sought to follow up on our observation of early Rtnl1 localization at the nuclear envelope and spindle poles at early prometaphase. We closely examined the localization of Rtnl1-GFP and RFP-KDEL during the transition from prophase to prometaphase during mitosis usingconfocal microscopy (Fig 2A). Low magnification images showed a brief but strong localization of Rtnl1-GFP at the poles prior to localization of RFP-KDEL. High magnification insets and precise time measurements during prometaphase showed that Rtnl1-GFP localized at the spindle poles 20 seconds prior to localization of RFP-KDEL (Fig 2B). We further quantitated this localization by measuring the mean and max fluorescence intensity of Rtnl1-GFP and RFP-KDEL at the poles (Fig 2C) as previously demonstrated in (Fig 1C and 1E –green dots) Intensity measurements were taken during interphase and measured throughout mitosis (time point 0–890 seconds). The start and timing of prometaphase was signaled by the breakdown of the nuclear envelope displayed by the disappearance of Lamin B (S1 Fig). Fluorescent intensity measurements of Rtnl1-GFP showed a steady increase and enrichment of intensity at the ROI between 0–540 seconds prior to NEB, (0.0102 slope 1, green line) while in contrast there is little increase of RFP-KDEL over that same time period (-0.0045 slope 1, red line). From time points 540–660 there is a dramatic increase of RFP-KDEL at the ROI (0.1116 slope 2, red line) compared to Rtnl1-GFP (0.06733 slope 2, green line). Here we conclude that Rtnl1 localization displays a steady enrichment at the spindle poles prior to the bulk accumulation of ER membrane early in mitosis.

### Proper localization of Rtnl1 depends on a dynamic microtubule network

**Fig 3:**
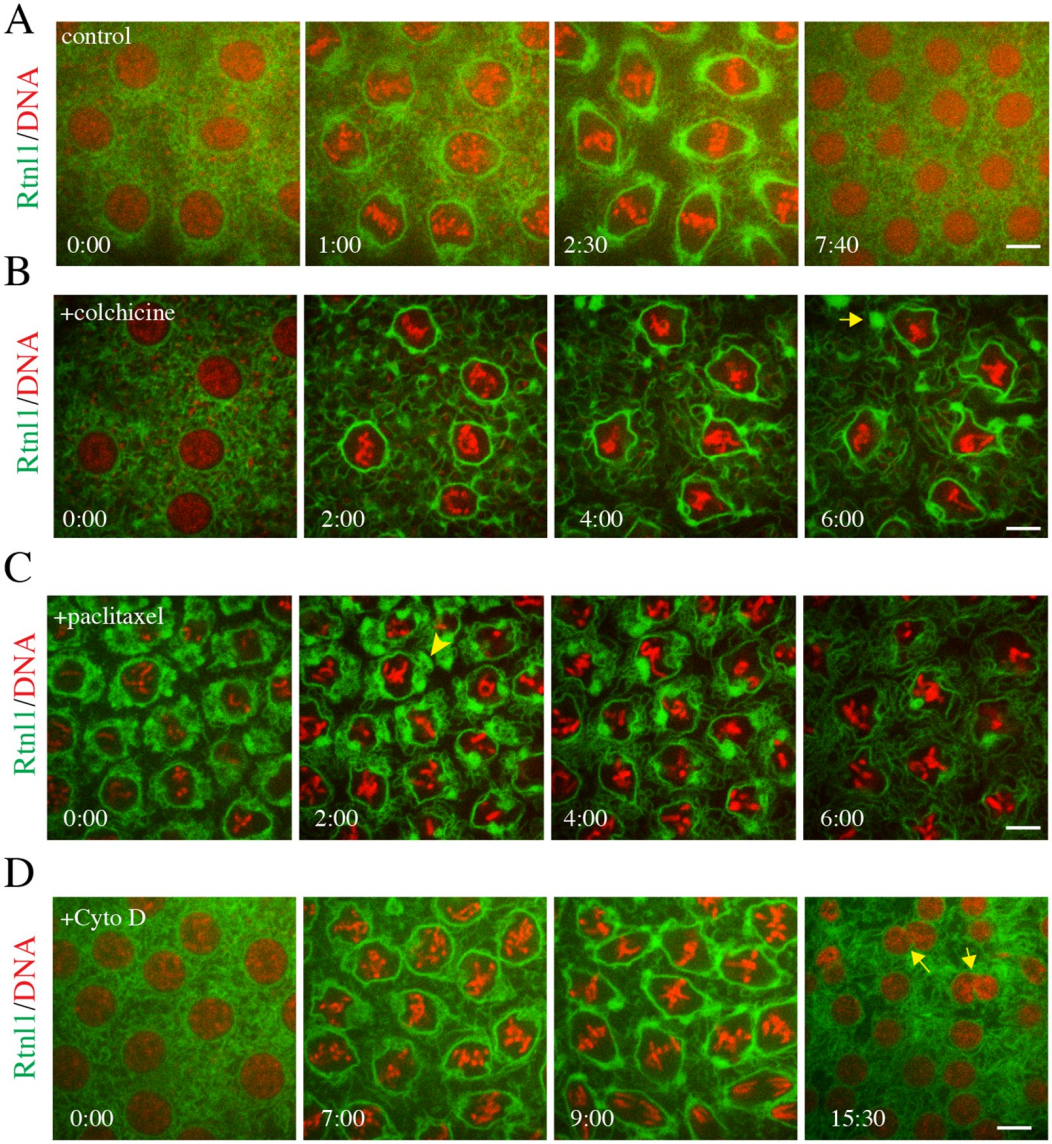
Proper localization of Rtnl1-GFP depends upon a dynamic MT network. **(A)** Control injection of a stage 10 Drosophila embryo expressing Rtnl1-GFP (green) and H2-RFP (red). Embryo was injected with 10% DMSO injection buffer. No defects were observed in ER localization or chromosome condensation and alignment and there was no delay in any stages of mitosis. (N = 6 embryos image) **(B)** Injection of colchicine 5 μM into a stage 9 embryo expressing Rtnl1-GFP and H2-RFP. Though chromosomes could condense, they did not align at metaphase, arresting the embryo. Rtnl1-GFP did gather at the nuclear periphery, but did not enrich at the poles. As the arrest continued, inter-nuclear ER became tubular and the ER at the nuclear periphery lost its even distribution, snapping back to large packets (yellow arrow). (N = 6 embryos imaged) **(C)** Injection of 5 μM paclitaxel into a stage 10 embryo expressing Rtnl1-GFP (green) and H2-RFP (red). Chromosomes condensed and aligned at the metaphase plate, but could not proceed, arresting the embryo. Rtnl1-GFP accumulated at the poles during the arrest (arrowhead). This accumulation was then lost as the arrest continued. N = 8 embryos imaged **(D)** Stage 9 embryo expressing Rtnl1-GFP (green) and H2-RFP (red) was injected with 5 μM Cytochalasin D to disrupt actin assembly. A lack of actin cages did not affect Rtnl1-GFP organization during mitosis or chromosome condensation and movement. However, at telophase, daughter nuclei that were within close proximity to each other fused (yellow arrows), creating large nuclei. N = 3 embryos imaged. Time is in min:sec. Scale bars are 10 μm.

Localization of Rtnl1 at the spindle poles prior to bulk ER may suggest that microtubules are important for ER membrane movement during mitosis. Previous studies have shown a requirement of the microtubule network for proper ER structure during interphase [15,16,36], yet, little is known about the role of microtubule dynamics on ER reorganization during mitosis. Additionally, the gradual change in Rtnl1 localization corresponds to changes in centrosome and microtubule dynamics during entry into mitosis suggesting an involvement of microtubules. To investigate the involvement of the microtubule network during mitosis, we employed temporally precise injections of small molecule microtubule dynamics inhibitors, just prior to the start of mitosis in the early syncytial embryo (Fig 3B and 3C). Use of these microtubule inhibitors has been well-documented in the early Drosophila embryo for rapid disruption of the microtubule network [37–39]. In our hands, these drugs affected mCherry-tubulin microtubules as predicted (S2 and S3 Figs). Rtnl1-GFP / H2-RFP embryos were injected with the microtubule depolymerizing agent colchicine just prior to entry into mitosis in cycle 10, after the nuclei have migrated to the cortex. Rtnl1-GFP was able to accumulate along the nuclear envelope, however at NEB, the ER membrane became disorganized and displayed severe clumping (Fig 3B arrow, S2 Fig, S2 Movie). Interestingly, the ER still remodeled into fenestrated mitotic clusters, however proper reorganization of the ER structures at the poles is severely disrupted. Injection of the microtubule stabilizing agent paclitaxel prior to entry into mitosis in cycle 11 initially did not affect the early reorganization of Rtnl1-GFP along the nuclear envelope, or accumulation at the poles (Fig 3C, arrowhead, S3 Fig). However, as mitosis progressed, Rtnl1-GFP organization destabilized at the spindle poles and the perispindle region. Treatment of either colchicine or paclitaxel caused a prolonged mitotic arrest at metaphase in which ER organization and structure were completely lost (Fig 3C, S2 and S3 Figs). Control injections of 10% DMSO (Fig 3A) did not display any defects in the microtubule network, mitotic progression, or ER reorganization.

### ER movement to the spindle pole is independent of the actin network

A previous study examining the remodeling and reorganization of the ER in the early *C*. *elegans* embryo suggested that the actin cytoskeleton mediated the mitotic reorganization and transitions of the ER [40]. In order to investigate the role of the actin network in mitotic Rtnl1 organization, we injected the actin polymerization inhibitor, Cytochalasin D (CytoD) just prior to entry into mitosis in cycle 11. CytoD has been shown to disrupt actin dynamics in the early Drosophila embryo [38,41,42]. In the presence of CytoD, Rtnl1 localization and ER distribution appeared normal with Rtnl1-GFP localizing to the spindle poles and along the perispindle region during prometaphase. CytoD did not cause a mitotic arrest, rather these embryos displayed severe defects in nuclear spacing and organization as previously published [43,44], which led to a few cases of nuclear fusion and fallout (arrow in Fig 3D). These results indicate that microtubule dynamics are necessary for proper reorganization of Rtnl1 at the spindle poles and along the perispindle region during mitosis independently of the actin cytoskeleton.

### Accumulation of Rtnl1 at the spindle poles early in mitosis relies on a dynamic astral microtubule network

Next, we investigated the role of astral microtubules in recruitment of Rtnl1 at the spindle poles upon entry into mitosis. There are several known Drosophila mutations that affect centrosome function and aster microtubule formation, however many of them affect genes that are maternally required for proper development of the syncytial blastoderm. Mutants known to reduce aster microtubules exhibit gross defects in nuclear spacing, chromosome segregation, spindle assembly and fail to develop to the stage when syncytial divisions begin [45,46]. To avoid these confounding effects, we used the small molecule inhibitors, Binucleine 2 and BI 2536 shown to be involved in maintaining astral microtubule populations.

Binucleine 2 is highly specific against isoforms of the serine/threonine kinase Aurora B in Drosophila [47]. Aurora B drives many events in mitosis including chromosome condensation and transmission [48,49]. Additionally, several studies have also linked Aurora B to mitotic spindle dynamics related to kinetochore microtubule attachment, spindle assembly checkpoint and the astral microtubule network [50,51]. To test the effects of Aurora B inhibition on ER accumulation to spindle poles during mitosis, we injected Binucleine 2 into Rtnl1-GFP / H2-RFP transgenic embryos just prior to entry into mitosis in cycle 10. Using spinning disk confocal microscopy, we imaged the dynamics of mitotic spindles in the presence of Binucleine 2, which displayed characteristic phenotypes attributed to loss of Aurora B in the Drosophila syncytial embryo including accumulation of free centrosomes and stunted and splayed spindle phenotype known to be associated with the loss of aster microtubules [52,53] (S4 Fig).

**Fig 4:**
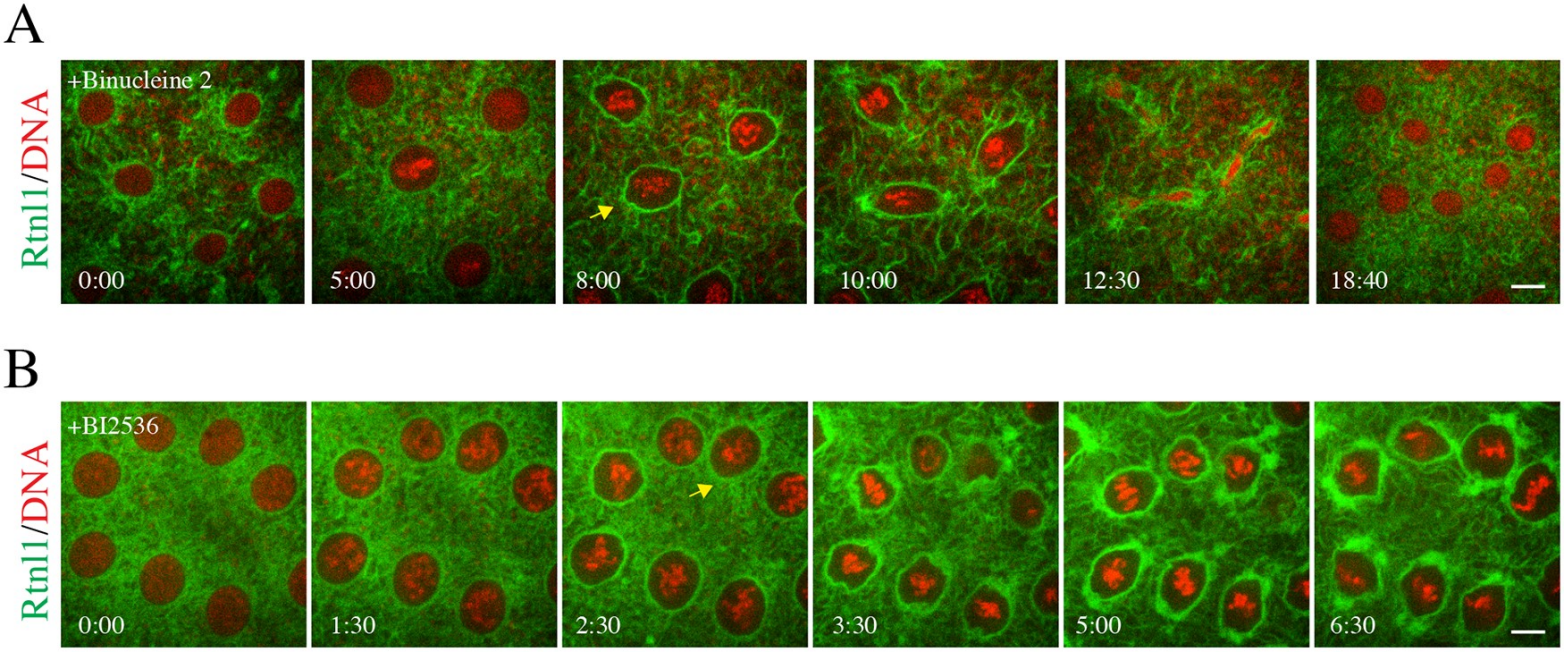
Rtnl1-GFP reorganization requires an astral microtubule network. **(A)** Stage 9 Drosophila embryo expressing Rtnl1-GFP (green) and H2-RFP (red) was injected with 10 μM Binucleine 2. Although the embryo proceeded through the next mitosis with a delay, several defects occurred. There was a lack of GFP accumulation at the poles until anaphase (yellow arrow). Telophase was also disturbed. N = 8 embryos imaged. **(B)** Injection of a stage 9 embryo expressing Rtnl1-GFP (green) and H2-RFP (red) with 10 μM BI 2536. This compound eventually arrested the embryo at a metaphase-like stage. Again, there was a lack of GFP accumulation before metaphase (yellow arrow). There was a greater than normal accumulation of Rtnl1-GFP at the nuclear periphery. N = 4 embryos imaged. Time is in min:sec. Scale bars are 10 μm.

Injection of Binucleine 2 did not affect reorganization and accumulation of Rtnl1-GFP at the nuclear envelope, however we observed a small lag in Rtnl1-GFP accumulation towards the spindle poles during prophase. (Fig 4A arrow). During the metaphase to anaphase transition, we observed a minor accumulation of Rtnl1 to spindle poles (Fig 4A 10:00 time point). Lastly, during telophase we also observed defects, including lagging chromosomes, and a lack of localization of Rtnl1 at the midbody (Fig 4A, 12:30 time point). Furthermore, these defects are consistent with well-established roles for inhibition of Aurora B, [54–56]. Because we found that Binucleine 2 eliminated astral microtubules but not spindle microtubules, these results suggest that localization of Rtnl1 at the spindle poles relies on astral microtubule dynamics.

In order to directly examine the role of astral microtubule populations in the accumulation of ER to spindle poles, we injected the Plk1 inhibitor, BI 2536 in Rtnl1-GFP / H2-RFP embryos just prior to entry into mitosis in cycle 10. Polo like kinase 1 (Plk1) is highly conserved and regulates many aspects of mitosis including centrosome maturation and migration, cohesion dissociation, and chromosome segregation [57]. Importantly, a recent study also showed that inhibition of Plk1 by BI 2536 specifically targets astral microtubule populations early in mitosis prior to metaphase [58]. Rtnl1-GFP displayed a strong accumulation to the nuclear envelope at prophase, however we observed a lack of accumulation at the centrosome at prometaphase (Fig 4B, arrow). As the embryo progressed into metaphase, we saw a gradual accumulation of Rtnl1-GFP to spindle poles. The embryo arrested at metaphase and there was a strong localization of Rtnl1-GFP at the poles and along the perispindle region. Previous studies involving the Plk1 inhibitor, BI 2536 displayed defects in centrosome migration and separation, astral microtubule formation at prophase and activation of the spindle assembly checkpoint [58,59]. The phenotypes observed in our analysis during mitosis are consistent with published roles of Plk1 [60] and the initial delay in localization of Rtnl1 at the spindle poles could be due to the lack of astral microtubules present early in mitosis. Based on our injections of Binucleine 2 and BI 2536, we conclude that Rtnl1 localization requires an active astral microtubule network.

### Inhibition of dynein does not affect spindle pole localization or distribution of mitotic ER at metaphase

**Fig 5:**
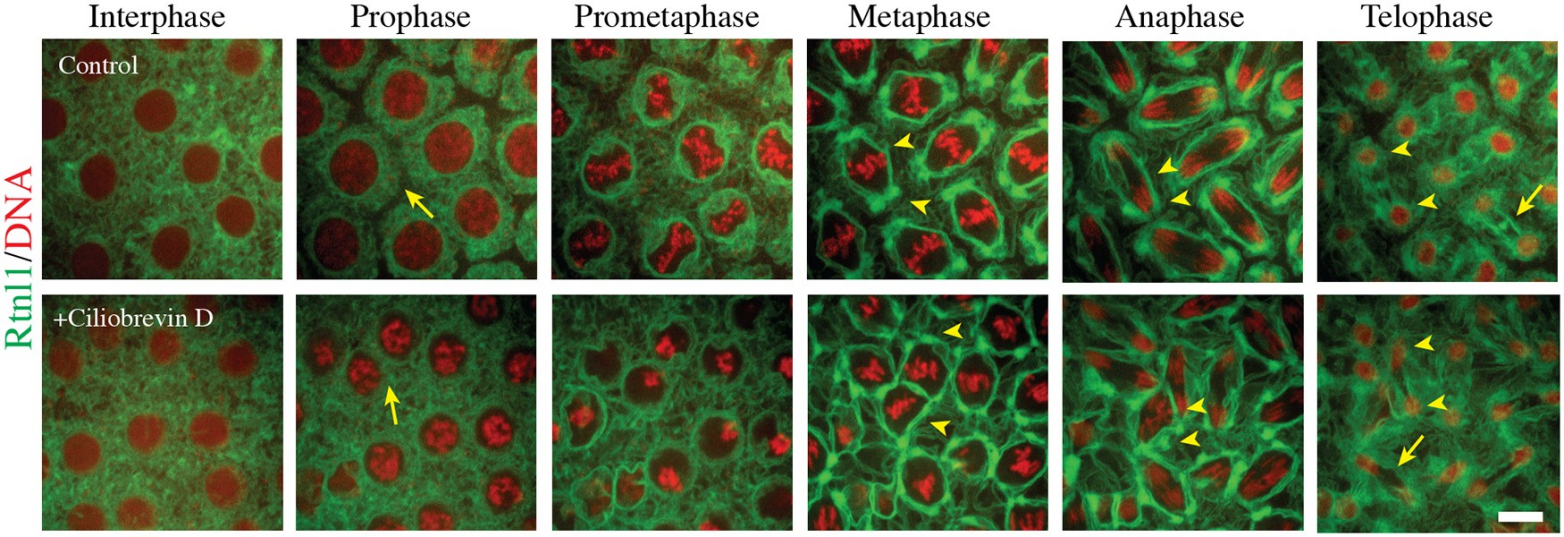
Rtnl1 accumulation at the spindle poles does not require dynein. Rtnl1-GFP (green); H2-RFP (red) transgenic embryos were injected with 100 μM Ciliobrevin D prior to entry into mitosis, cell cycle 10 and imaged at cell cycle 11. Initially there are defects in accumulation of ER towards the nuclear envelope prior to nuclear envelope breakdown (NEB) (arrow). After NEB, the ER is able to localize along the mitotic spindle and spindle poles (arrowheads) at metaphase through anaphase. During telophase, the ER is able to be partitioned at cytokinesis in the presence of Ciliobrevin D (arrowheads), however there is a defect with localization of ER at the midbody with presence of Ciliobrevin D (arrow). Scale bars are 10 μm. N = 5 embryos imaged.

The movement of Rtnl1 towards the spindle poles during mitosis suggests a role for the minus-end directed microtubule motor dynein in this reorganization. Several studies have implicated dynein in the localization of endosomes and Golgi vesicles at the centrosome [61–63]. In addition, *in vitro* Xenopus egg extract experiments implicated cytoplasmic dynein being responsible for ER localization along the mitotic spindle [64]. In order to examine the role of cytoplasmic dynein on Rtnl1-GFP localization at the centrosome, we injected Ciliobrevin D in the early Drosophila embryo and examined the effects on Rtnl1 localization along the perispindle region and at the centrosomes. Ciliobrevin D has been shown to inhibit cytoplasmic dynein by interfering in the conversion of chemical energy into mechanical force specific to the minus-end director motor [65]. In addition, a recent study showed Ciliobrevin D blocked dynein function in the developing Drosophila embryo and greatly affected endosome populations and intracellular vesicular trafficking [66]. In order to examine the efficacy of Ciliobrevin D inhibition of cytoplasmic dynein during the syncytial divisions, we injected tubulin-GFP / H2-RFP transgenic embryos and examined mitotic spindle assembly for any defects related to dynein (S5 Fig). Upon exposure to Ciliobrevin D, we observed classic defects of tripolar spindle formation (S5 Fig, arrowheads), including free centrosomes and spindle pole focusing which are consistent with the inhibition of dynein in the early embryo [33,67]. Injection of Ciliobrevin D in Rtnl1-GFP / H2-RFP embryos, just prior to the start of mitosis in cycle 11, led to defects in nuclear spacing and accumulation of Rtnl1-GFP at the nuclear envelope during prophase (Fig 5 Arrows, S3 Movie). However, after nuclear envelope breakdown, Rtnl1-GFP was able to localize to the perispindle region and the spindle poles during metaphase and persisted through anaphase and telophase (Fig 5 arrowheads). Also observed was a lack of Rtnl1-GFP localization at the midbody in the presence of Ciliobrevin D at telophase (Fig 5 Arrow). Furthermore, there were incidences of accumulation of Rtnl1 localization away from the spindle, however we believe this is due to the presence of free centrosomes, a defect attributed to inhibition of dynein [33,68].

To confirm our observation involving dynein’s lack of involvement in ER localization at the mitotic spindle poles, we imaged mitotic embryos that expressed double-stranded RNA against the dynein heavy chain (DHC-RNAi) and transgenic RFP-KDEL (S6 Fig). Similar to Ciliobrevin D, in the presence of the DHC-RNAi, there was a large presence of free centrosomes, centrosomes not associated with a nucleus or mitotic spindle, and tripolar spindles [69,70]. Early in mitosis, there was a lack of accumulation of ER membrane at the nuclear envelope. Interestingly, both free centrosomes and tripolar spindle showed a strong localization and reorganization of ER membrane (S6 Fig). Taken together these qualitative results suggest that dynein does not affect the bulk recruitment of Rtnl1 or ER to the spindle poles or the presence of Rtnl1 at free centrosomes. These data further suggest that a separate factor associated with the spindle poles is involved in the recruitment of ER membrane.

### Quantitation of Rtnl1 and ReepB movement at the spindle poles in the presence of small molecule inhibitors

In order to employ a quantitative approach to measure ER enrichment to spindle poles, we developed an algorithm to manually segment and track ROIs on spindles and in the cytoplasm just prior to and during mitosis. This script, developed in MATLAB, allows for the bulk extraction of measurements for multiple ROIs across a series of images. Following bulk extraction, we used particle tracking to assemble data for individual spindle and cytoplasm ROIs. Lastly, we normalized data to represent intensity fold change from time 0 for individual spindle and cytoplasm ROIs. Although we plotted spindle and cytoplasm ROIs together, they represent data for two different sets of ROIs taken from the same image.

**Fig 6:**
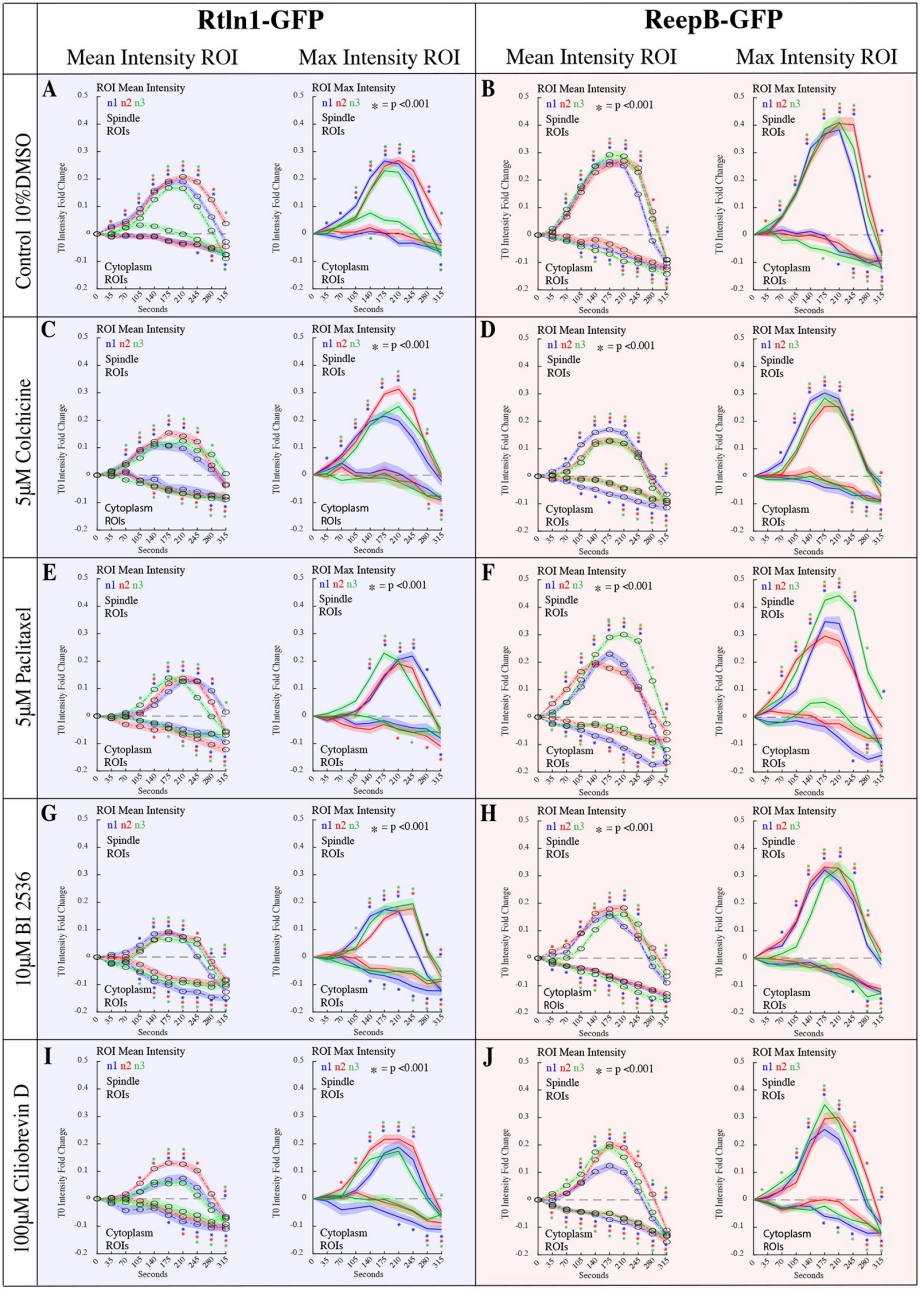
Quantitation of Rtnl1-GFP and ReepB-GFP enrichment at the spindle poles during mitosis. Four our quantitative approach, we track 20 spindles and 20 cytoplasm ROIs across 10 frames for 3 injected embryos per treatment (A-J). Spindle and cytoplasm ROIs were placed as depicted in (Fig 1C and 1E). In total we measure 6000 ROIs, to produce a total of 12,000 measurements (A-J). **(A)** 10% DMSO control injections for Rtnl1-GFP. Control embryos show a 0.1 fold increase in enrichment (p<0.001 for all samples at T210) compared to non-injected samples (Fig 1D) (p<0.001 for all samples at T210). **(B)** ReepB-GFP embryos injected with 10% DMSO to serve as control. Control embryos show a 0.1 fold increase in enrichment (p<0.001 for all samples at T210) compared to non-injected samples (Fig 1E; p<0.001 for all samples at T210). **(C)** Rtnl1-GFP embryos injected with 5 μM Colchicine. Here we measure a reduction in Rtnl1-GFP enrichment to spindles (p<0.001 for all samples at T210) compared to controls (A) (p<0.001 for all samples at T210). **(D)** ReepB-GFP embryos injected with 5 μM Colchicine. We measure a reduction in ReepB-GFP enrichment to spindles (p<0.001 for all samples at T210) compared to controls (B) (p<0.001 for all samples at T210). **(E)** Rtnl1-GFP embryos injected with 5 μM paclitaxel. **(F)** ReepB-GFP embryos injected with 5 μM paclitaxel. This treatment looks similar to the control 10% DMSO results (B) **(G)** Rtnl1-GFP embryos injected with10 μM BI 2536. We measure a reduction in Rtnl1-GFP enrichment to spindles (p<0.001 for all samples at T210) compared to controls (A) (p<0.001 for all samples at T210). **(H)** ReepB-GFP embryos injected with10μM BI 2536. We measure a reduction in ReepB-GFP enrichment to spindles (p<0.001 for all samples at T210) compared to controls (B) (p<0.001 for all samples at T210). **(I)** Rtnl1-GFP embryos injected with100 μM Ciliobrevin D. We measure a reduction in Rtnl1-GFP enrichment to spindles (p<0.001 for all samples at T210) compared to controls (A) (p<0.001 for all samples at T210). **(J)** ReepB-GFP embryos injected with100 μM Ciliobrevin D. We measure a reduction in ReepB-GFP enrichment to spindles (p<0.001 for all samples at T210) compared to controls (B) (p<0.001 for all samples at T210).

Here, we repeated the temporally precise small molecule injections, similar to the experiments (Figs 3–5) into Rtnl1-GFP and ReepB-GFP embryos and used a quantitative approach to measure enrichment to spindles and depletion from the cytoplasm. We found that injections of 10% DMSO, our control treatment (Fig 6A and 6B), produced a 0.1 fold enrichment increase compared to non-injected embryos (Fig 1D and 1F), including a slight lag in Rtnl1-GFP cytoplasmic depletion. We note that 10% DMSO control treatment bares the most resemblance to paclitaxel treatment (Fig 6F and 6G), possibly highlighting the known microtubule stabilizing effects of DMSO [71]. Furthermore, injections of colchicine (Fig 6C and 6D) into Rtnl1-GFP and ReepB-GFP resulted in a lower fluorescence intensity of these proteins at the poles compared to 10% DMSO controls, suggesting that microtubule assembly facilitates ER enrichment to spindles. Here we note that in colchicine treatment, ReepB-GFP is slightly more affected than Rtnl1-GFP (Fig 6C and 6D). Moreover, injections of the Plk1 inhibitor BI 2536 also showed a lower fluorescence intensity at spindle poles; however, here Rtnl1-GFP enrichment was reduced more than ReepB-GFP enrichment. Lastly, injections of the dynein inhibitor, Ciliobrevin D, into Rtnl1-GFP and ReepB-GFP embryos displayed an identical intensity pattern as compared to BI 2536 treatment (Fig 6H and 6I), suggesting that aster microtubules and dynein influence Rtnl1-GFP more so than ReepB-GFP. Although our qualitative data showed that Rtnl1-GFP was enriched to spindle poles during mitosis in Ciliobrevin D treatment (Fig 5, S6K Fig), our quantitative approach showed that less Rtnl1-GFP made it to spindle poles, even though more Rtnl1-GFP was depleted from the cytoplasm (Fig 6I). Additionally, in the presence of Ciliobrevin D treatment Rtnl1-GFP appeared to be enriched around the nucleus more so than in controls (Fig 5, S6K Fig). A greater depletion from the cytoplasm with less enrichment to spindles could be explained by greater enrichment around the nuclear envelope with a failure to move towards spindles as mitosis progresses.

## Discussion

Research over the last decade has highlighted the dramatic changes of the ER during cell division, however the factors that govern these mitotic changes are poorly understood [7,11,68]. This study has focused on the dynamics of the highly conserved ER shaping protein, Rtnl1, during mitosis. Here we show that Rtnl1 displays a steady enrichment at the nuclear envelope and spindle poles prior to bulk of the ER membrane at prometaphase. Using precise temporal inhibition, we show that microtubule dynamics are necessary for proper ER localization and partitioning during mitosis. Furthermore, the small molecule inhibitors, Binucleine 2 and BI 2536, which affect the formation of astral microtubules, leads to defects in ER localization at the spindle poles early in mitosis. Blocking cytoplasmic dynein both by small molecule injection and RNAi does not affect localization of Rtnl1 at the poles. This work highlights the mechanistic requirements of mitotic ER localization and provides a framework for ER partitioning during cell division.

A general concern with the approach of using small molecule inhibitors to examine mitotic ER organization is that they can be broad acting and the defects observed can be attributed to indirect or downstream disruptions of the cell cycle. However, we believe that the phenotypes observed for Rtnl1 are direct outcomes of disruption of the cytoskeletal networks. This is in large part to a prior study that showed when the cell cycle was arrested using small molecule inhibitors either in interphase or mitosis, ER structure initially was unaffected and only after 15–20 minutes of arrest, were any ER defects observed [11]. The ER defects shown in this study were immediate, within 1–2 minutes after exposure to the small molecule indicating a more direct role.

### Movement of ER membrane during mitosis requires a dynamic microtubule network

Research over the last decade has elucidated that the ER is a dynamic organelle drastically changing its shape and localization upon entry into mitosis [7,8,72]. The mitotic factors responsible for these dramatic changes involving the ER remains an area of great interest. It has been well established that organization of the ER relies on the microtubule network during interphase [15,16,36] allowing the ER to stretch from the nuclear envelope to the cell periphery, however it is unknown if the microtubule network performs a similar organizational role during mitosis. Studies involving ER movement during mitosis in *S*. *cerevisiae* and *C*. *elegans* have implicated the actin cytoskeleton network in mitotic ER dynamics [40,73]. The ER shares a close localization with the mitotic spindle and poles and it has generally been thought that microtubules and associated motor proteins are responsible for mitotic ER organization and partitioning [26]. To this end, an investigation into the ER transmembrane protein, STIM1 showed an interaction with the microtubule plus-end tracking protein (+TIP) EB1 [74]. Additionally, this interaction between the ER and microtubule network is regulated by phosphorylation of STIM1 [75]. However, sequence analysis between the mammalian STIM1 and the isoforms of the Drosophila homolog, dSTIM1, showed that dSTIM1 does not contain the identified EB1 binding site or the serine or threonine regulatory amino acids, indicating the existence of multiple mechanisms for ER / microtubule interactions. In support of the role of microtubules, there have been studies implicating the astral microtubule network in partitioning of the ER during asymmetric neuroblast divisions [68].

Our data, as well as previous published reports, display a strong localization of ER at the spindle poles during mitosis (Figs 1 and 2) [11,76]. It has been assumed that ER is transported towards the poles by the major cellular minus-end directed motor, dynein. In support of this, cytoplasmic dynein has been implicated in trafficking and transport through the secretory system, as well as in the structural support and localization of organelles during interphase [77,78]. It is also well established that dynein is involved in several mitotic processes including nuclear envelope breakdown through pulling forces along the astral microtubule network, thereby leading to bipolar spindle formation at metaphase [79,80]. In addition, dynein has also been implicated in the transport and localization of recycling endosomes at the spindle poles during early mitosis [33]. We show that small molecule inhibition of dynein during mitosis, while disrupting proper spindle assembly, did not prevent ER localization at the spindle poles (Fig 5). This qualitative result of dynein independence is in line with a very recent study showing that dynein does not affect ER movement to the spindle poles in Drosophila spermatocytes [81]. This result suggests that ER is not being transported or maintained at the minus-end of the microtubules by dynein. However, our quantitative approach suggests that a small amount of Rtnl1 movement is affected and largely lags along the nuclear envelope (Fig 6I). While this lack of movement was shown to be significant, this can be explained by the known role of dynein involvement in the breakdown of the nuclear envelope [79] thereby affecting timing of NEB and release of the mitotic kinase Cyclin A affecting ER reorganization [11] rather than a direct connection of dynein with Rtnl1.

Gurel et al. [77], in a review focused on ER calcium sequestration suggested two models of microtubule-based ER transport, the sliding mechanism and / or the tip attachment complex (TAC). Sliding mechanism focuses on motor-based movement along an existing microtubule, while TAC model suggest that ER structural proteins would attach to a +TIP protein and movement would be connected to microtubule growth. While there is evidence in different systems for each, our data suggest that there is a direct connection to microtubule dynamics of growth and stability of the ER. Furthermore, based on our small molecule inhibition of the astral microtubule network, this suggests that astral microtubule dynamics also are key in proper mitotic localization of ER at the poles (Fig 4). Recently, a study also showed that the kinesin-14 family member of microtubule minus-end directed motor protein, non-claret disjunctional (ncd) or the kinesin 5 plus-end directed microtubule motor protein Klp61F did not affect ER movement to the spindle poles [81]. Future studies should investigate other possible candidate proteins including the astral microtubule associated kinesin motor protein Khc-73 [82] or the NUMA ortholog, Mushroom body defective (Mud) [83] that associate with the spindle poles or along the astral microtubule network.

### The reticulon family of ER shaping proteins are candidates for mitotic ER movement and partitioning during cell division

Early investigations into the mechanism that regulates ER shape and structure identified a class of proteins, known as Reticulons [19,84]. This protein family is not defined by sequence homology, but rather by the presence of two short hairpin transmembrane domains on the cytoplasmic leaflet of the ER [85]. Recent studies have begun to elucidate the role of these Reticulon family members in both regulating the structural changes of the ER and connection to the cytoskeleton [86,87]. Rtnl1 was the first reticulon family member identified in Drosophila, however several additional proteins with reticulon homology domains (RHD) have recently been identified in Drosophila and other systems including spastin, atlastin-1, DP1/YOP and REEPs [24,88]. It has been shown that these proteins can oligomerize and form homomeric and heteromeric complexes in regards to ER tubule formation [20]. Recently, the mammalian REEP3/4 proteins have been shown to contribute to membrane curvature changes in mitosis and interact with microtubules [25]. However, it is unclear if the REEP proteins work in concert with the reticulons during mitosis. Here, our data demonstrates the importance of the microtubule network in organizing mitotic ER. Furthermore, we believe that our disruption of the mitotic spindle and ER organization is a direct outcome and not a downstream consequence of affecting mitotic progression, as a previous study in the early embryo showed that ER dynamics are in frame with the cell cycle and were halted when cell cycle was blocked. However, even with these disruptions, the ER maintained its mitotic organization [11]. Future directions regarding mitotic ER organization would be to identify additional targets that regulate ER organization and partitioning during mitosis. An interesting candidate is the microtubule severing enzyme, spastin. Several studies have indicated an interaction between reticulons and spastin [88,89], however, spastin has recently been shown to mediate contacts between the ER and lysosomes [90]. Future studies that investigate the biochemical interaction between the reticulon family members and the role that mitotic regulatory factors play in complex formation should provide insight into mitotic ER dynamics.

### Quantitative analysis of ER membrane movement during mitosis

Much of the research in cell biology focused on analysis of fixed or live images to explore and understand cellular function. However, this analysis has largely relied on the individual selecting the region of analysis, thereby giving a qualitative overview of any given phenotype. Furthermore, this type of qualitative analysis, while useful, is difficult to compare with predictions from *in silico* modeling [91–93]. Moreover, high content screening efforts require numerical measures in order to allow statistical analysis of results and identification of hits. While automated high-content image analysis has been extensively employed in studies of mammalian cells in culture, quantitative and automated methods remain under-utilized in studies of the Drosophila embryo. In order to address a quantitative approach with respect to ER movement along the perispindle region and spindle poles during mitosis, we developed a MATLAB code that allowed for the unbiased selection of ROIs at spindle poles in the syncytial embryo (Fig 6A and S4 Fig) and applied this to high-content data collected with Rtnl1-GFP and ReepB-GFP embryos injected with different small molecule inhibitors (Fig 6B–6K). This quantitative approach gave similar results that were in line with our qualitative injection analysis (Figs 3–5), with some minor exceptions. Seemingly contrary to our qualitative observation of embryos injected with the cytoplasmic dynein inhibitor, Ciliobrevin D (Fig 5), our quantitative analysis (Fig 6I) reveals less Rtnl1-GFP enrichment to spindles, and more Rtnl1-GFP depletion from the cytoplasm. An observation for this discrepancy between observations and measurements is that Rtnl1-GFP is enriched around the nuclear envelope but fails to move toward spindles upon NEB and as mitosis progresses, which is apparent in Fig 5 and S6K Fig. Similarly, our quantitative approach showed a decrease in ReepB-GFP enrichment to spindles with an increase in cytoplasmic depletion in Ciliobrevin D treatment (Fig 6J), (S6J Fig). Furthermore, our use of a qualitative and quantitative analysis demonstrates the strength of using a mixed method approach which we believe will greatly advance the field by providing key insights to the mechanistic underpinnings of complex cellular processes.

## Supporting information

S1 Fig Stage 10 GFP-Lamin embryo imaged through mitosis. GFP-Lamin localized along the nuclear periphery at the start of mitosis. At 5:20 the nucleus destabilized indicating the start of nuclear envelope breakdown (NEB), by 9:20 timepoint Lamin disappeared from the nucleus and reappeared at 15:00 at cytokinesis and nuclear envelope reformation. Time is in min:sec. Scale bars are 5 μm.
(TIF)

S2 Fig Injection of colchicine depolymerizes the mitotic spindle. Rtnl1-GFP / mCherry-Tub embryo was injected with 5μM colchicine just prior to the start of mitosis of cycle 11. As the embryo enters mitosis, the nuclear envelope broke down and initially the mitotic spindle formed. Rtnl1-GFP localized to the spindle poles and along the perispindle region. As colchichine took effect, the spindle slowly dissolved and Rtnl1-GFP localization along the spindle area is deformed. N = 3 embryos imaged. Time is in min:sec. Scale bars are 5 μm.
(TIF)

S3 Fig Injection of paclitaxel stabilizes the mitotic spindle. Paclitaxel was injected into Rtnl1-GFP / mCherry Tubulin embryo just prior to the start of mitosis of cycle 11. As the embryo progressed through mitosis, the mitotic spindle formed and Rtnl1-GFP reorganized to the spindle poles and perispindle region. In the presence of paclitaxel, the spindle stabilized and the embryo arrested at metaphase. Rtnl1-GFP maintained its localization at the spindle poles and the perispindle region. N = 4 embryos imaged. Time is in min:sec. Scale bars are 5 μm.
(TIF)

S4 Fig Injection of Binucleine 2 displayed defects in astral microtubule formation. Rtnl1-GFP / mCherry Tubulin embryo was injected with 10 μM Binucleine 2 just prior to the start of mitosis of cycle 11 and imaged through metaphase of cycle 12. In the presence of Binucleine 2, initially mitotic events proceed normally, as asters and centrosomes can be viewed (arrowheads) and Rtnl1-GFP localization appeared normal. However, as the embryo moved into mitosis cycle 12, aster and spindle formation were affected including stunted and splayed spindles (arrows) and the presence of tripolar spindles indicating that astral microtubule network was disrupted. N = 4 embryos imaged. Time is in min:sec. Scale bars are 5 μm.
(TIF)

S5 Fig Injections of Ciliobrevin D displays classic defects attributed to dynein inhibition. Ciliobrevin D was injected into GFP-tubulin (green); H2Av-RFP (red) stage 10 embryos just prior to entry into mitosis. Defects including formation of tripolar nuclei (arrow), tripolar spindles and fee centrosomes (arrowheads) have been previously attributed to inhibition of cytoplasmic dynein in the early Drosophila embryo [52]. Scale bars are 5 μm.
(TIF)

S6 Fig Expression of DHC RNAi in the early Drosophila embryo does not affect ER localization at the spindle poles. Dynein Heavy Chain (DHC) RNAi line was expressed in UAS-RFP-KDEL embryos. Images were taken from cycle 10 prophase to cycle 11 metaphase. Defects, including tripolar spindle and free centrosomes were present (arrowheads). ER was still able to localize to defective spindles and centrosomes. Scale bars are 5 μm.
(TIF)

S7 Fig Quantitation of Rtnl1-GFP and ReepB-GFP enrichment at the spindle poles during mitosis. Embryos were microinjected at cell cycle 7 then imaged from cell cycle 10–13. (A) 60x field of view of ReepB-GFP during mitosis in cell cycle 10. The blue square represents the field of view used for panels B-K. Panel A is processed with a threshold binary filter. This filter is not applied to Panels B-K since it excludes data from pixels below the set threshold. (B, D, F, H, J) ReepB-GFP in control, colchicine, paclitaxel, BI 2536, and Ciliobrevin D. (C, E, G, I, K) Rtnl1-GFP in control, paclitaxel, BI 2536, and Ciliobrevin D. Images for ReepB-GFP across all conditions were captured using the same exposure. Images for Rtnl1-GFP across all conditions, except Ciliobrevin D (K), were captured using a different exposure. Panel K was accidentally captured with exposure for Reep-GFP. (D) colchicine shows a decreased enrichment of ReepB-GFP compared to controls.
(TIF)

S8 Fig Quantitative analysis pipeline. To measure enrichment at spindle poles and depletion from the cytoplasm, we developed a custom MATLAB script to facilitate the collection and analysis of 6000 ROIs from 30 microinjected embryos. We designed our script to implement manual segmentation followed by automated extraction and assembly of data for individual ROIs across a 10-frame series of images. We start by turning Z stacks into max intensity projections (B, C). Max intensity projections are shown to a user 1 at a time for segmentation (D). Using a binary mask, the program turns user defined segmentations into jpegs (E), which can be used to extract additional values for the same ROIs at any future time. As the program generates jpegs, it produces a binary matrix that defines ROI locations (F). Once the user has segmented all frames, the binary matrix is applied to the original series of images and ROI mean and max intensity values, along with ROI [x, y] coordinates, are bulk extracted from each frame (G). ROI values and coordinates are stored in a new matrix (H). Using a custom particle tracking algorithm, the program identifies ROIs that correspond to each other across frames (I). Using index values stored in a separate matrix (J) the program assembles ROI values across time in a new Matrix (K). Lastly, the program normalizes for T0 fold change and saves values using a file naming system that feeds directly into a custom complier script used to put together graphs for experimental groups (L). One iteration of the program produces data for one line (M) and one complete graph requires 6 iterations. (N). Data for one experimental group requires 12 iterations (O).
(TIF)

S1 Movie Rtnl1-GFP localization and dynamics during the syncytial divisions in the early embryo. Transgenic embryo expressing Rtnl1-GFP (green) and H2-RFP (red) from cycle 10 to cycle 13. Frames were taken every 30 seconds. Time is min:sec. Scale bar is 10 μm.
(MOV)

S2 Movie Injection of colchicine displays defects in proper Rtnl1 localization. Transgenic embryo expressing Rtnl1-GFP (green) and H2-RFP (red) injected with 5 μM colchicine prior to the start of mitosis in cycle 11. Frames were taken every 30 seconds. Time is min:sec. Scale bar is 5 μm.
(MOV)

S3 Movie Injection of Ciliobrevin D does not affect Rtnl1 localization at the spindle poles. Transgenic embryo expressing Rtnl1-GFP (green) and H2-RFP (red) injected with 100 μM ciliobrevin D prior to the start of mitosis in cycle 11. Frames were taken every 30 seconds. Time is min:sec.
(AVI)
