# Supplementary figures and images for: Microtubules are necessary for proper Reticulon localization during mitosis
Source: PLoS One. 2019 Dec 26;14(12):e0226327. doi: 10.1371/journal.pone.0226327 (PMC6932760; doi:10.1371/journal.pone.0226327)

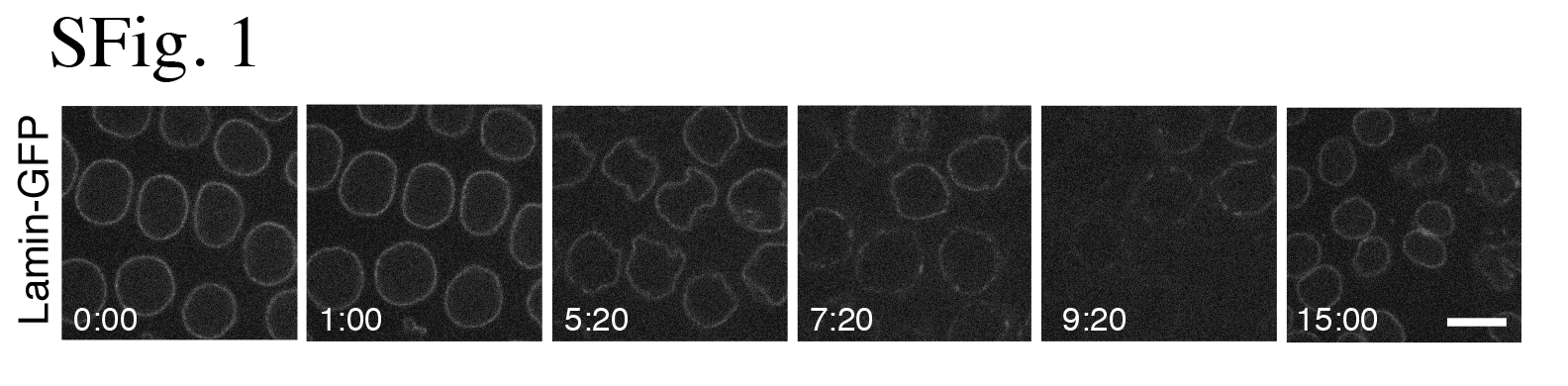

Supplement: S1 Fig — GFP-Lamin localized along the nuclear periphery at the start of mitosis. At 5:20 the nucleus destabilized indicating the start of nuclear envelope breakdown (NEB), by 9:20 timepoint Lamin disappeared from the nucleus and reappeared at 15:00 at cytokinesis and nuclear envelope reformation. Time is in min:sec. Scale bars are 5 μm. (TIF) [file pone.0226327.s001.tif]

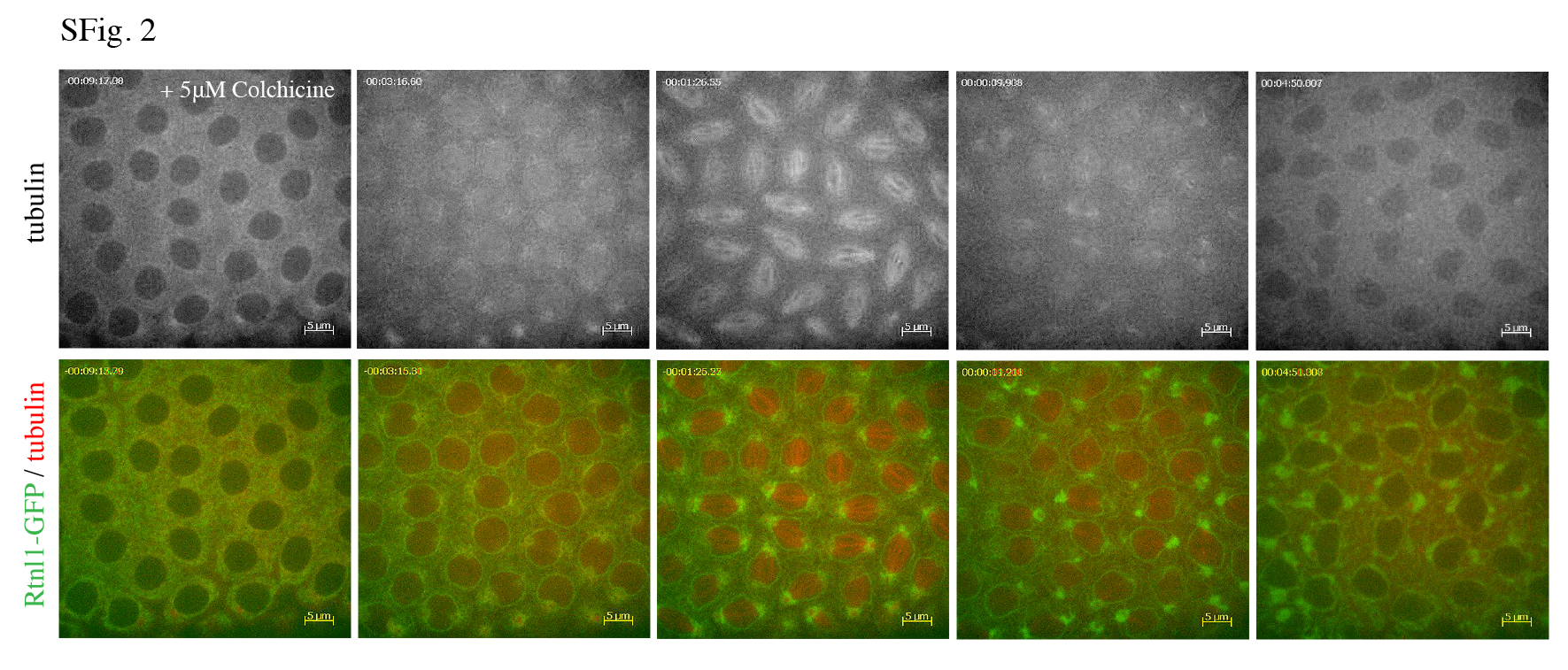

Supplement: S2 Fig — Rtnl1-GFP / mCherry-Tub embryo was injected with 5μM colchicine just prior to the start of mitosis of cycle 11. As the embryo enters mitosis, the nuclear envelope broke down and initially the mitotic spindle formed. Rtnl1-GFP localized to the spindle poles and along the perispindle region. As colchichine took effect, the spindle slowly dissolved and Rtnl1-GFP localization along the spindle area is deformed. N = 3 embryos imaged. Time is in min:sec. Scale bars are 5 μm. (TIF) [file pone.0226327.s002.tif]

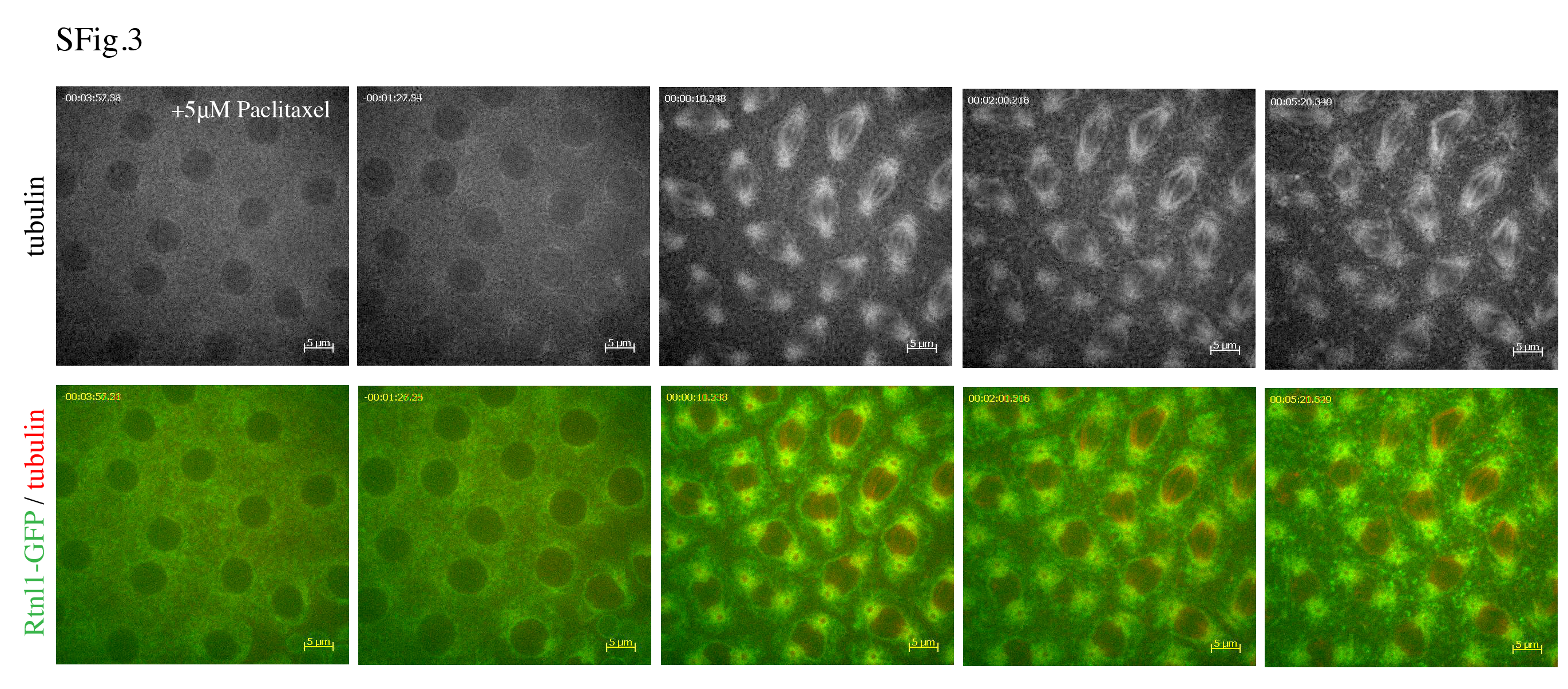

Supplement: S3 Fig — Paclitaxel was injected into Rtnl1-GFP / mCherry Tubulin embryo just prior to the start of mitosis of cycle 11. As the embryo progressed through mitosis, the mitotic spindle formed and Rtnl1-GFP reorganized to the spindle poles and perispindle region. In the presence of paclitaxel, the spindle stabilized and the embryo arrested at metaphase. Rtnl1-GFP maintained its localization at the spindle poles and the perispindle region. N = 4 embryos imaged. Time is in min:sec. Scale bars are 5 μm. (TIF) [file pone.0226327.s003.tif]

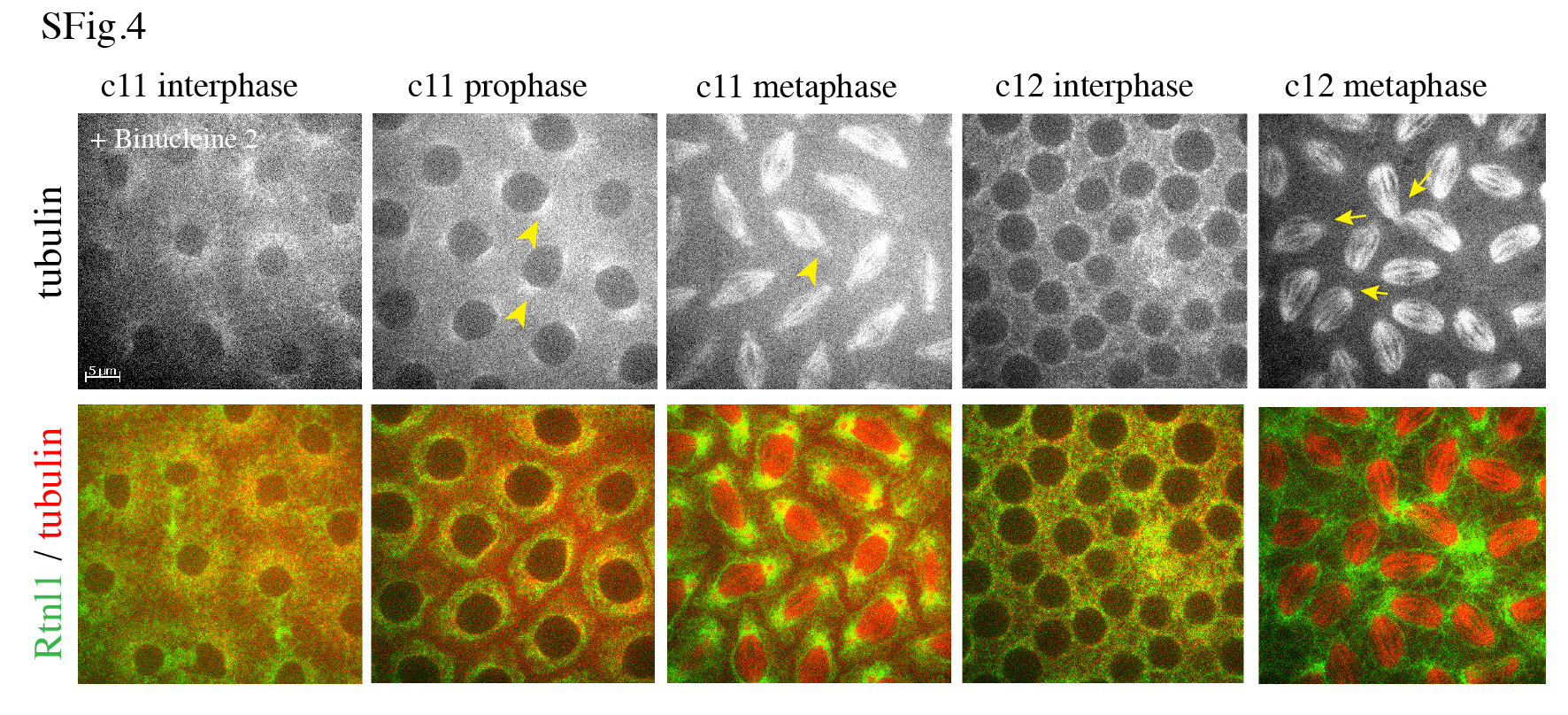

Supplement: S4 Fig — Rtnl1-GFP / mCherry Tubulin embryo was injected with 10 μM Binucleine 2 just prior to the start of mitosis of cycle 11 and imaged through metaphase of cycle 12. In the presence of Binucleine 2, initially mitotic events proceed normally, as asters and centrosomes can be viewed (arrowheads) and Rtnl1-GFP localization appeared normal. However, as the embryo moved into mitosis cycle 12, aster and spindle formation were affected including stunted and splayed spindles (arrows) and the presence of tripolar spindles indicating that astral microtubule network was disrupted. N = 4 embryos imaged. Time is in min:sec. Scale bars are 5 μm. (TIF) [file pone.0226327.s004.tif]

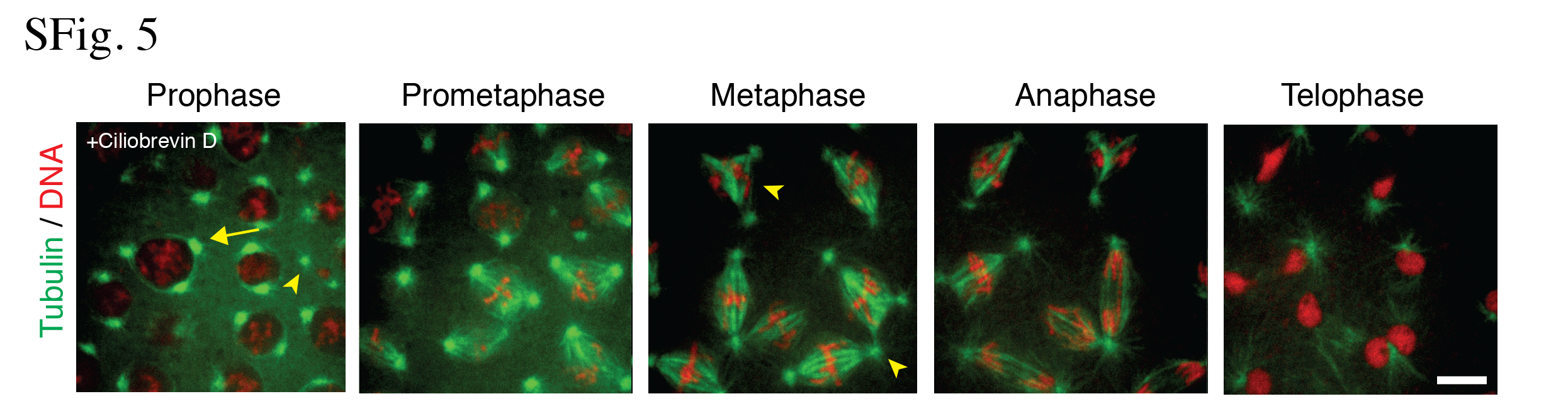

Supplement: S5 Fig — Ciliobrevin D was injected into GFP-tubulin (green); H2Av-RFP (red) stage 10 embryos just prior to entry into mitosis. Defects including formation of tripolar nuclei (arrow), tripolar spindles and fee centrosomes (arrowheads) have been previously attributed to inhibition of cytoplasmic dynein in the early Drosophila embryo [52]. Scale bars are 5 μm. (TIF) [file pone.0226327.s005.tif]

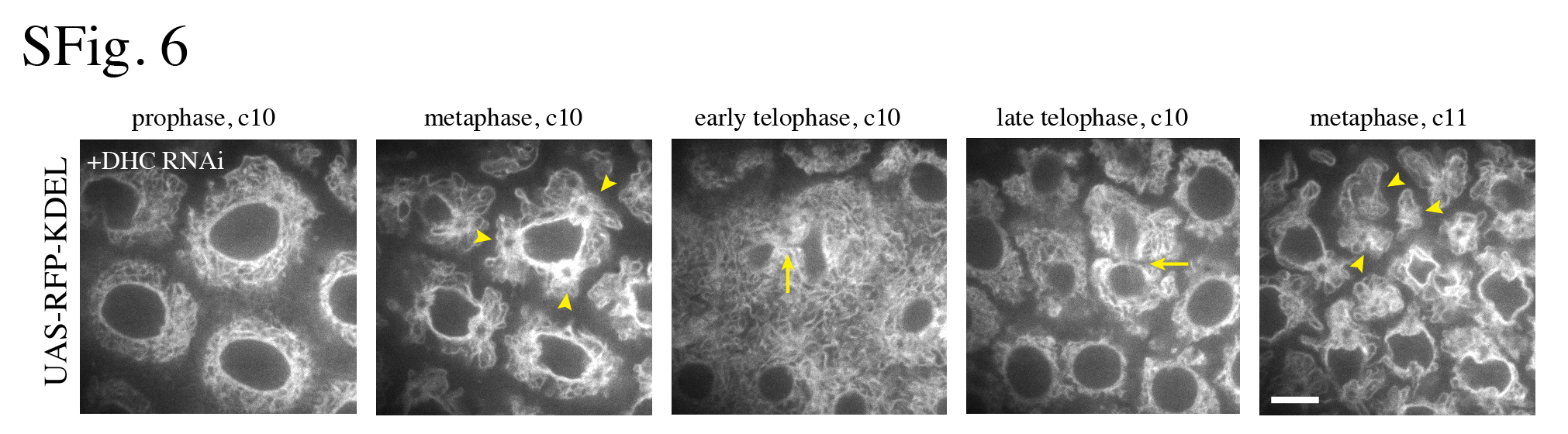

Supplement: S6 Fig — Dynein Heavy Chain (DHC) RNAi line was expressed in UAS-RFP-KDEL embryos. Images were taken from cycle 10 prophase to cycle 11 metaphase. Defects, including tripolar spindle and free centrosomes were present (arrowheads). ER was still able to localize to defective spindles and centrosomes. Scale bars are 5 μm. (TIF) [file pone.0226327.s006.tif]

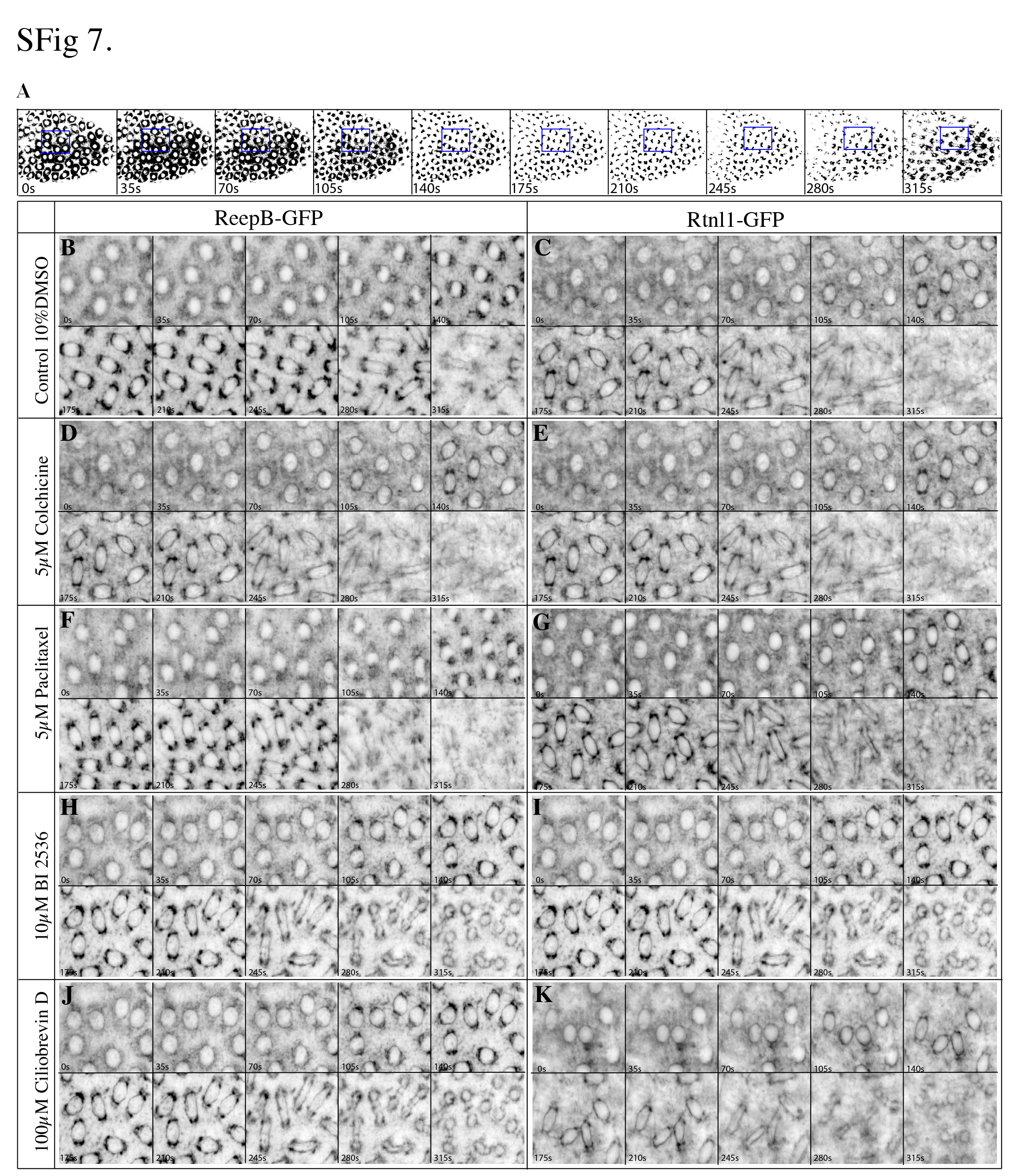

Supplement: S7 Fig — Embryos were microinjected at cell cycle 7 then imaged from cell cycle 10–13. (A) 60x field of view of ReepB-GFP during mitosis in cell cycle 10. The blue square represents the field of view used for panels B-K. Panel A is processed with a threshold binary filter. This filter is not applied to Panels B-K since it excludes data from pixels below the set threshold. (B, D, F, H, J) ReepB-GFP in control, colchicine, paclitaxel, BI 2536, and Ciliobrevin D. (C, E, G, I, K) Rtnl1-GFP in control, paclitaxel, BI 2536, and Ciliobrevin D. Images for ReepB-GFP across all conditions were captured using the same exposure. Images for Rtnl1-GFP across all conditions, except Ciliobrevin D (K), were captured using a different exposure. Panel K was accidentally captured with exposure for Reep-GFP. (D) colchicine shows a decreased enrichment of ReepB-GFP compared to controls. (TIF) [file pone.0226327.s007.tif]

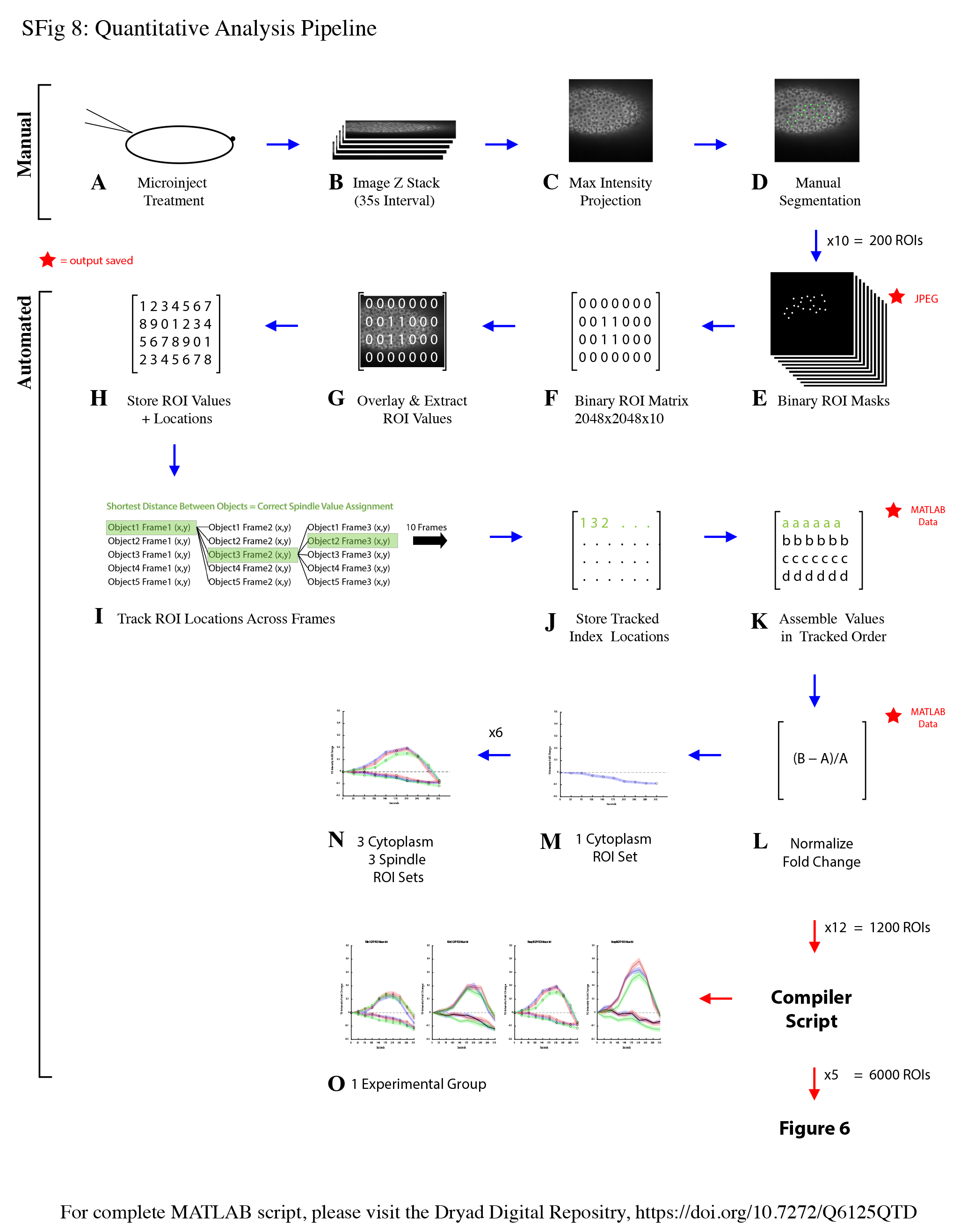

Supplement: S8 Fig — To measure enrichment at spindle poles and depletion from the cytoplasm, we developed a custom MATLAB script to facilitate the collection and analysis of 6000 ROIs from 30 microinjected embryos. We designed our script to implement manual segmentation followed by automated extraction and assembly of data for individual ROIs across a 10-frame series of images. We start by turning Z stacks into max intensity projections (B, C). Max intensity projections are shown to a user 1 at a time for segmentation (D). Using a binary mask, the program turns user defined segmentations into jpegs (E), which can be used to extract additional values for the same ROIs at any future time. As the program generates jpegs, it produces a binary matrix that defines ROI locations (F). Once the user has segmented all frames, the binary matrix is applied to the original series of images and ROI mean and max intensity values, along with ROI [x, y] coordinates, are bulk extracted from each frame (G). ROI values and coordinates are stored in a new matrix (H). Using a custom particle tracking algorithm, the program identifies ROIs that correspond to each other across frames (I). Using index values stored in a separate matrix (J) the program assembles ROI values across time in a new Matrix (K). Lastly, the program normalizes for T0 fold change and saves values using a file naming system that feeds directly into a custom complier script used to put together graphs for experimental groups (L). One iteration of the program produces data for one line (M) and one complete graph requires 6 iterations. (N). Data for one experimental group requires 12 iterations (O). (TIF) [file pone.0226327.s008.tif]
